# Percutaneous Breast Biopsy: Local Tumor Spread, Ontogenetic Compartments, and Cell Transgression/Reverse Morphogenesis Theory

**DOI:** 10.7759/cureus.82504

**Published:** 2025-04-18

**Authors:** Eduardo De Faria Castro Fleury, Marcia G Tavares, Veronica J Ayres

**Affiliations:** 1 Radiology, MD duFLE Diagnósticos, São Paulo, BRA; 2 Nuclear Medicine, Instituto Brasileiro de Controle do Câncer, São Paulo, BRA; 3 Gynecology, Faculdade de Medicina do ABC, Sao Paulo, BRA

**Keywords:** breast cancer, morphogenesis, needle biopsy, ontogenetic anatomy, post-biopsy change, tumor prognostic factors

## Abstract

Background

Observing patients submitted to percutaneous biopsy at our service who had post-biopsy breast magnetic resonance imaging scans for breast carcinoma staging, we observed tumor extension through the biopsy tract in some lesions, compromising skin and subcutaneous tissue. This study aimed to evaluate tumor extension through the biopsy tract in patients diagnosed with breast carcinoma undergoing neoadjuvant chemotherapy with a radioactive seed marker to locate the original tumor area by retrospectively analyzing MRI, ultrasonography, and histological reports.

Methods

We evaluated tumor extension through a biopsy tract in consecutive patients included in a prospective observational study protocol to assess the applicability of radioactive seed localization before breast surgery. Sixty-two patients included in the radioactive seed protocol were retrospectively evaluated. The abnormal enhancement in the biopsy tract was assessed by magnetic resonance imaging scans performed for clinical staging and seed localization as proposed in the original study protocol, with complementary target ultrasonography of the findings and ultrasound-guided biopsy of the suspected lesion.

Results

Four of the 62 patients in the protocol had abnormal enhancement in the biopsy tract, and three patients had a positive biopsy for carcinoma in the tract. The lesions that showed extension along the tract were luminal A, luminal B, and luminal hybrid carcinomas, with an average diameter of 3.9 cm.

Conclusions

Our study has shown the possibility of tumor extension through the biopsy tract in patients undergoing diagnostic percutaneous breast biopsy for breast cancer. These findings are crucial for understanding the biopsy procedures’ potential risks and clinical implications.

## Introduction

Percutaneous breast biopsies are part of the diagnosis, staging, and therapeutic strategy of breast cancer treatment. There is no consensus on the best technique and number of specimens collected during the procedure. The needles used for biopsy vary between 8 G and 16 G, attached to automatic, semi-automatic, or vacuum-assisted devices. Some studies speculate that neoplastic cells could spread through the biopsy tract by cell seeding, but without scientific proof of the pathophysiology of this tumor extension [1,2,3].

Observing patients referred to a post-biopsy breast MRI scan for clinical staging in our institution, we observed tumor extension through the biopsy tract in some of them, with extension to the skin and subcutaneous tissue confirmed by biopsy.

Following the theory of the ontogenetic compartment for tumor dissemination in breast cancer, the tumor should respect each duct-lobular system derived from a primordial mammary epithelium through morphogenesis. However, invasion occurs through reverse morphogenesis when the cancer spreads to a similar and adjacent ontogenetic compartment. Otherwise, transgression occurs when the tumor extends to a neighboring compartment of a different embryological origin. Both transgression and reverse morphogenesis are preceded by focal inflammation and fibrosis [4].

The medical literature does not appear to agree on the prevalence and pathophysiology of tumor spread along the biopsy path [1-3]. In this article, we discuss the impact of percutaneous biopsies on local tumor spread following these theories in patients included in a study protocol for radioactive seed localization before breast surgery, assuming that biopsy favors tumor cells' dissemination into the biopsy tract, which is favored by the inflammatory process inherent to the procedure. We also discussed the possible impact of breast biopsies on the tumor’s microenvironment and alternatives to improve the biopsy technique.

This study primarily aims to evaluate tumor extension through the biopsy tract in patients diagnosed with breast carcinoma undergoing neoadjuvant chemotherapy by retrospectively analyzing MRI scans.

## Materials and methods

We retrospectively evaluated MRI scans, ultrasonography, and histological and immunohistochemical reports of patients included in a prospective observational study protocol for evaluating radioactive seed localization before breast surgery from 01.02.2019 to 10.06.2020. The main objective of the initial study was to prospectively assess the efficiency of preoperative localization using radioactive seeds in patients diagnosed with breast cancer and MRI cancer staging. After the seed placement, the patients underwent preoperative MRI to topograph the radioactive seed localization. The interval between the biopsy and the MRI was up to two months following our institution’s workflow.

The MRI protocol was standard, using a MAGNETOM Espree 1.5 T echo scanner (Siemens, Munich, Germany) with a specific 8-channel breast coil. The protocol consists of axial sequences weighted in T1-weighted with fat suppression, axial short-tau inversion recovery (STIR), and each unilateral sagittal sequence in proton density (PD).

The contrast media (gadolinium) was administered to all patients without contraindication to the paramagnetic agent for further investigation. The dynamic axial sequences were acquired before and after contrast (gadobenate dimeglumine, MultiHance, Bracco) administration for 4 min. All images were reconstructed in the axial plane with subtraction and maximum intensity-projection (MIP) techniques.

A radiologist with 8 years’ experience in breast imaging read the MRI findings. During the interpretation process, the reader attempted to identify the site and tract of the percutaneous biopsy, from the skin and subcutaneous tissue to the periphery of the tumor. Patients whose tract was visible on MRI were recalled for targeted ultrasonography.

Another radiologist with 20 years of experience in breast imaging performed the target ultrasonography. Ultrasonography was performed using a Toshiba Aplio 300 ultrasound system (Canon Medical Systems Corporation, Ōtawara, Tochigi, Japan) with a 5-14 mHz multifrequency linear probe. When the tract corresponded with ultrasonography, a biopsy was performed for histopathological confirmation.

The same radiologist performed the biopsy of the suspected area using a 14-G needle with a semi-automatic device, BARD Core Magnum (Bard Company, New Providence, New Jersey, USA), with multiple insertions and at least two samples collected. The needle incursion of 2.2 cm was adopted for all biopsies. The radiologist targeted the center of the lesion due to the small dimensions of the tract impairment. The radiologist also evaluated the specimen’s hardness, color, and dimensions to ensure the quality of the material. The material was considered suitable when the fragment appeared hard in consistency, in a MonoBlock, white/grayish color, and immersed in a formalin bottle.

The biopsy samples were sent to the institution’s Pathology Department for histological and immunohistochemical evaluation.

After the histological and immunohistochemical results were available, the radiologist correlated them with the imaging findings to validate the results. When the results were in agreement, the radiologist considered the procedure successful.

All patients participating in the initial study to evaluate radioactive seed localization before breast surgery were included in the analysis, and their data were evaluated retrospectively. Exclusion criteria consisted of patients who did not undergo a complementary MRI study after the biopsy and those who underwent neoadjuvant chemotherapy between the biopsy and MRI, since neoadjuvant chemotherapy response could mask the tumor extension along the biopsy path.

Based on the biopsy results, we determined the frequency of MRI biopsy tract involvement, the correlation of MRI findings with ultrasound, and the presence of malignant cells in the tract. In addition, we evaluated which histological type was prevalent in tract involvement.

The data used in this study were obtained through retrospective analysis of MRI and second-look ultrasound reports and histological and immunohistochemical results of percutaneous biopsies of patients participating in the initial prospective protocol.

The results were analyzed statistically using MedCalc for Windows version 19.4 (MedCalc Software, Ostend, Belgium). The statistical summary of the quantitative numerical variables was obtained, and a one-sample t-test was performed, with significance levels of p<0.05.

This retrospective study was approved by the institutional ethics committee and, with an informed consent form signed by the patients, was registered on the Brazil Platform under “Certificado de Apresentação de Apreciação Ética” (CAAE): 45979021.9.0000.0072.

## Results

Between February 1, 2019, and June 06, 2020, 62 patients underwent radioactive seed localization before breast surgery to mark primary breast cancer. All 62 patients filled the inclusion criteria. The average time between the diagnostic biopsy and the staging MRI was 45 days.

According to histology, the most prevalent tumor in the study was luminal B (21 of 62 patients, 33.9%), followed by triple-negative (16 of 62 patients, 25.8%), luminal hybrid (12 of 62 patients, 19.4%), luminal A (9 of 62 patients, 14.5%), while the least prevalent was HER2(+). The mean measurement of the lesions included in the study was 36.0 mm (varying from 18 mm to 100.00 mm, with 95% IC of 31,2255 to 41,5023), where HER2-positive tumors had the smallest mean size of 27.5 mm (varying from 20.4 mm to 32.6 mm, with 95% IC of 11,7570 to 42,7430), while triple-negative tumors were the largest, with a mean of 39.7 mm (varying from 21 mm to 80.3 mm, with 95% IC of 26,6385 to 49,0447). The mean age of the patients involved in the study was 52 years, where hybrid luminal tumors were prevalent in the youngest patients (41 years). In comparison, triple-negative tumors affected the oldest patients (58.5 years).

Table 1 shows the patients’ ages and lesion dimensions according to the immunochemistry type of the participants in the protocol.

**Table 1:** Patients’ ages and lesion dimensions according to the immunochemistry type of the participants in the protocol.

		Luminal A	Luminal B	HER2(+)	Luminal Hybrid	Triple-Negative	Total
N		9	21	4	12	16	62
(%)		14.5%	33.9%	6.5%	19.4%	25.8%	100.0%
Dimension (mm)	Median	36.0	35.4	27.5	36.4	39.7	36.0
	Average	38.6	37.6	27.0	42.2	31.7	38.8
	SD	13.0	13.2	5.1	22.7	22.7	15.6
Age (year)	Median	53.0	49.0	53.0	41.0	58.5	52
	Average	37.0	49.7	41.0	47.2	38.6	51.2
	SD	8.9	9.7	6.6	18.1	11.1	10.5

Four (6.4%) of the 62 patients were diagnosed with a biopsy tract impairment on MRI. All MRI-positive findings could be observed at second-look ultrasonography, with three (75.0%) testing positive for malignancy.

**Figure 1:**
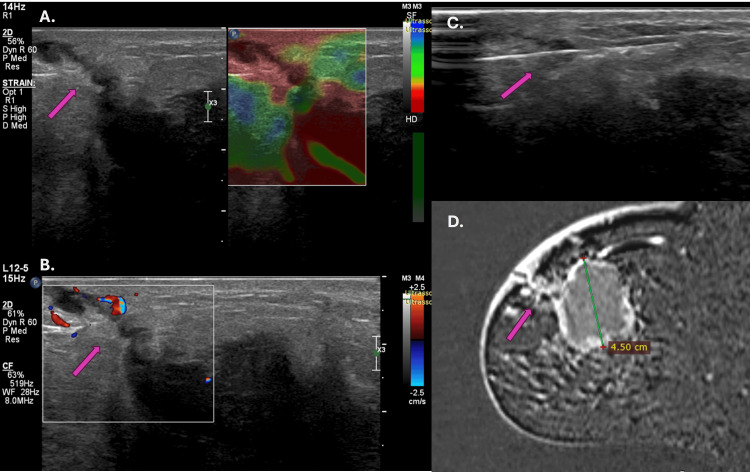
Ultrasonography with elastography (A), color Doppler study (B), percutaneous biopsy (C), and magnetic resonance imaging, (D) of a lesion in the biopsy tract in luminal A carcinoma. The arrow shows the lesion in the needle tract (A, B, C, and D). A hypoechogenic lesion with a peripheral halo, rigid on elastography (A) and with increased vascularization (B), extends from the mass surface to the skin and subcutaneous tissue. Biopsy of the lesion with the needle in the center (C). Post-contrast MRI showing the extension to the skin and subcutaneous tissue (D).

Table 2 shows the MRI-positive findings of the biopsy tract impairment according to age, dimension, immunochemistry type, and KI-67 results (Table 2). The histological types from biopsy specimens were luminal A (Figure 1), luminal B, and luminal hybrid. The KI-67 test ranged from 5 to 30%, as shown in Table 2. The negative biopsy result shows macrophages, lymphocytes, and giant cell granulomas with foreign body reactions at histology. 

**Table 2:** The MRI-positive findings of the biopsy tract impairment according to age, dimension, immunochemistry type, and KI-67 results.

	Age (Year)	Dimension (mm)	Type	KI-67
1	48	40	Luminal B	20
2	46	34	Luminal A	5
3	40	41	Luminal hybrid	30

## Discussion

Neoplastic seeding of the biopsy tract following percutaneous needle biopsy is controversial in the literature. Despite being well-documented for some types of cancer, such as hepatocellular carcinoma, non-small cell lung carcinoma, renal cell carcinoma, and thyroid carcinoma, the pathophysiology of this dissemination is not well established [1-3]. Some authors assume that the displacement of epithelial cells is responsible for this dissemination. Brenner et al. reported a 0.8% rate of neoplastic implantation [5]. Santiago et al. defined neoplastic seeding as malignant-appearing masses or calcifications that developed along the biopsy needle tract extending to subcutaneous tissues and skin following the needle biopsy, not present on imaging before or at the time of the breast biopsy. In the article published in 2017, 4010 patients diagnosed with breast cancer were retrospectively evaluated, and eight patients had a diagnosis of neoplastic seeding. The average time of appearance was 60.8 days [3].

**Figure 2:**
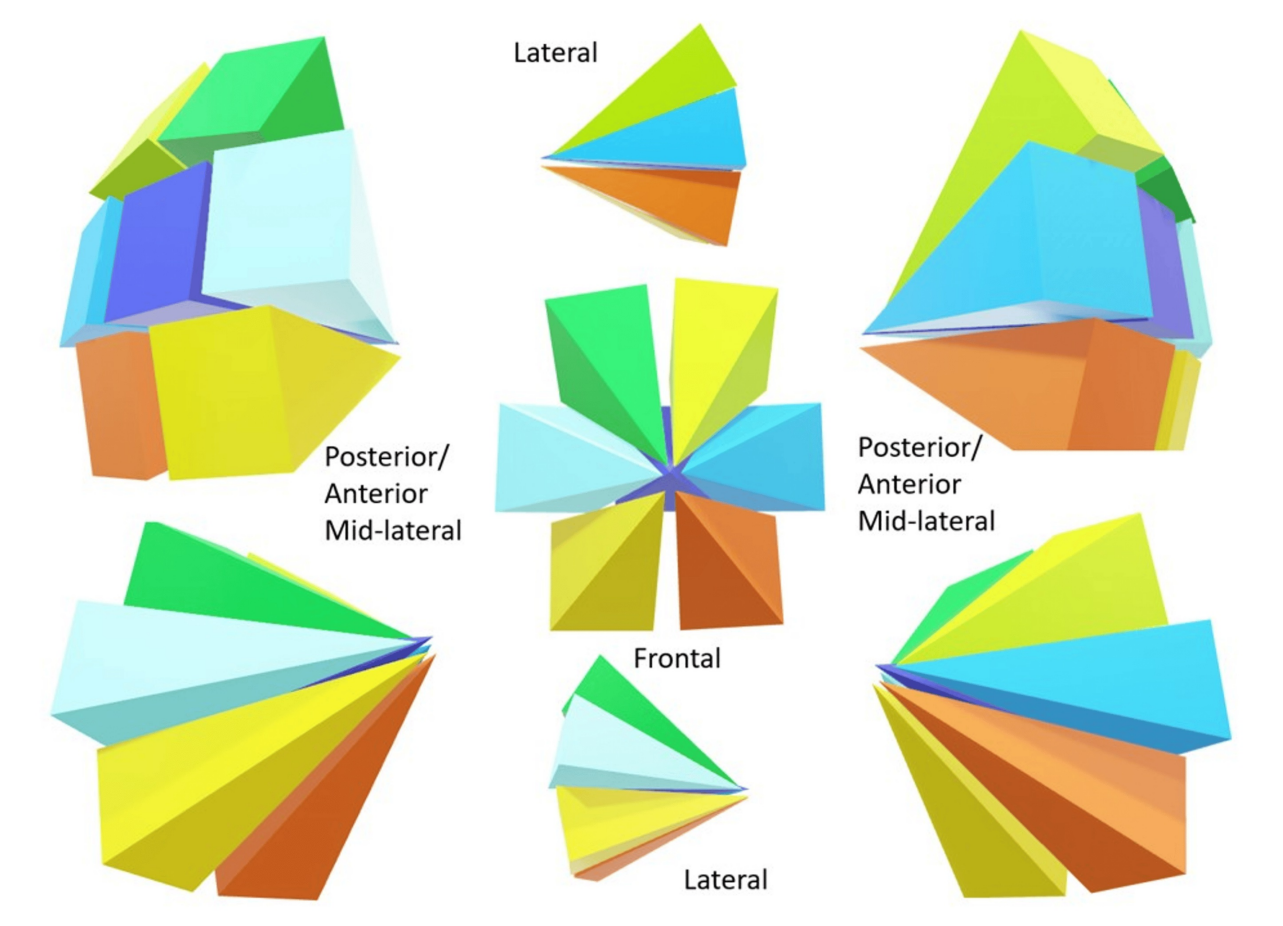
Schematic illustration of the ontogenetic compartments of the breast. Each ontogenetic compartment is compatible with a mammary lobe, containing duct-lobular units that do not communicate with the other compartments (each different colored cone). Image credits: Eduardo de Faria Castro Fleury.

Some concepts are essential to understanding neoplastic seeding. The female breast is formed by 15-20 lobular duct units with a shared origin in the areola-papillary complex. Embryologically, each duct-lobular unit of the breast represents a distinct subcompartmental anatomy originating from an individual sprout of the breast epithelial primordium, denominating an ontogenetic compartment (Figure 2) [6,7]. An article on “Reflection and Reaction,” published by Hockel in 2009, discusses the importance of the ontogenetic compartment: “For conservative resection of DCIS and early invasive cancer lesions, the margins at the circumferential border of the compartment can be close without the risk of local recurrence. Intracompartmental margins should be substantially wider to rule out occult residual disease”. In the article, the author also argues that there is clinical evidence that wound-healing reaction from cancer resection stimulates the initiation and growth of the local relapse of malignant lesions and might also enhance its malignant progression, possibly through a hypoxic environment, resulting in more aggressive disease [6].

The local dissemination of invasive carcinoma is an isotropic process of tissue infiltration independent of tumor margins, according to Hockel in another article published in 2012 [8]. The tumor microenvironment favors invasion of the interstitium, intravasation of lymphatic and venous channels, and perineural spread. The edges of the home compartment suppress tumor growth. The author stated that each breast lobe unit corresponds to a distinct ontogenetic compartment, where tumor dissemination is more favored toward this compartment's interior than the periphery. The author postulates that tumor invasion into another ontogenetic compartment is favored by a local inflammatory process associated with tumor cells' reverse morphogenesis or transgression. Functional differences are also observed between tumors extending into the same compartment and infiltrating a neighboring compartment. Tumors infiltrating the adjacent compartment are well-oxygenated, while those confined to the ontogenetic space are hypoxic [8].

In 2023, McCarty et al. published “Sustained Inflammation of Breast Tumors Affecting Needle Biopsy” [9]. According to the authors, performing a biopsy triggers a wound-healing response and a foreign-body reaction. Inflammation, hemorrhage, fat necrosis, granulation tissue, necrosis, and giant cell reaction are common findings in the biopsy tract, as well as atypical spindle cells and atypical duct-like structures have also been reported. The primary tumor cells in the biopsy tract have been observed in some cases. The wound-healing process consists of four phases: hemostasis, inflammation, proliferation, and remodeling. The inflammatory phase predominates neutrophils and eosinophils, followed by macrophages that release cytokines and chemokines. The authors demonstrate that the prolonged presence of macrophages and eosinophils slows down the wound-healing process, regardless of the histological pattern of the tumor. In the study, the authors demonstrate the presence of macrophages in the biopsy site for an extended period (average 34+/- 28.8 days). The transition of macrophage phenotypes for tissue repair follows from M0 - M1 - M2. The M2 phenotypes involved in the wound-healing process and the tumor-associated macrophages (TAMs) are classified differently but have similar actions mediating cell proliferation and angiogenesis through cytokines, chemokines, and growth factors. As M2 TAMs are related to a worse prognosis, it would be essential to understand the M2/M1 relationship after biopsy and the possible alterations in neighboring cells. In other words, there is ambiguous information for the defense system regarding the difference between repairing a tissue by wound-healing and protecting the host from tumor dissemination [9].

Another peculiar factor described by McCarty is the presence of neutrophils, eosinophils, and plasma cells carrying biopsy marker material. Theoretically, the biopsy localization markers are wrapped in biocompatible, biodegradable material to prevent displacement. This degraded material found in inflammatory cells in the biopsy site may prolong the inflammatory process. Our study used titanium capsules with radioactive iodine-125 as a biopsy marker. The influence of these devices on the tumor microenvironment is not established [9].

We followed up with 62 consecutive patients subjected to consecutive placement of radioactive markers in the biopsy site, where three of the 62 patients (4.8%) showed tumor seeding along the biopsy tract within 2 months. The tumor dissemination seeding was higher in our study compared to Santiago et al., which may be explained by the study methodology, where the patients who underwent biopsy were followed up during the surgical planning interval in a prospective study. Another factor that may have contributed is the use of MRI for tumor evaluation, the most sensitive diagnostic method for detecting breast cancer.

As the extension in the biopsy tract are new lesions and have the potential to change clinical/surgical management, associated with 4.8% of malignancy through the biopsy tract in our results, means that according to the Breast Imaging and Reporting Data System (BI-RADSTM) lexicon fifth edition recommendations, these findings should be classified as suspicious and included in category 4 [10].

**Figure 3:**
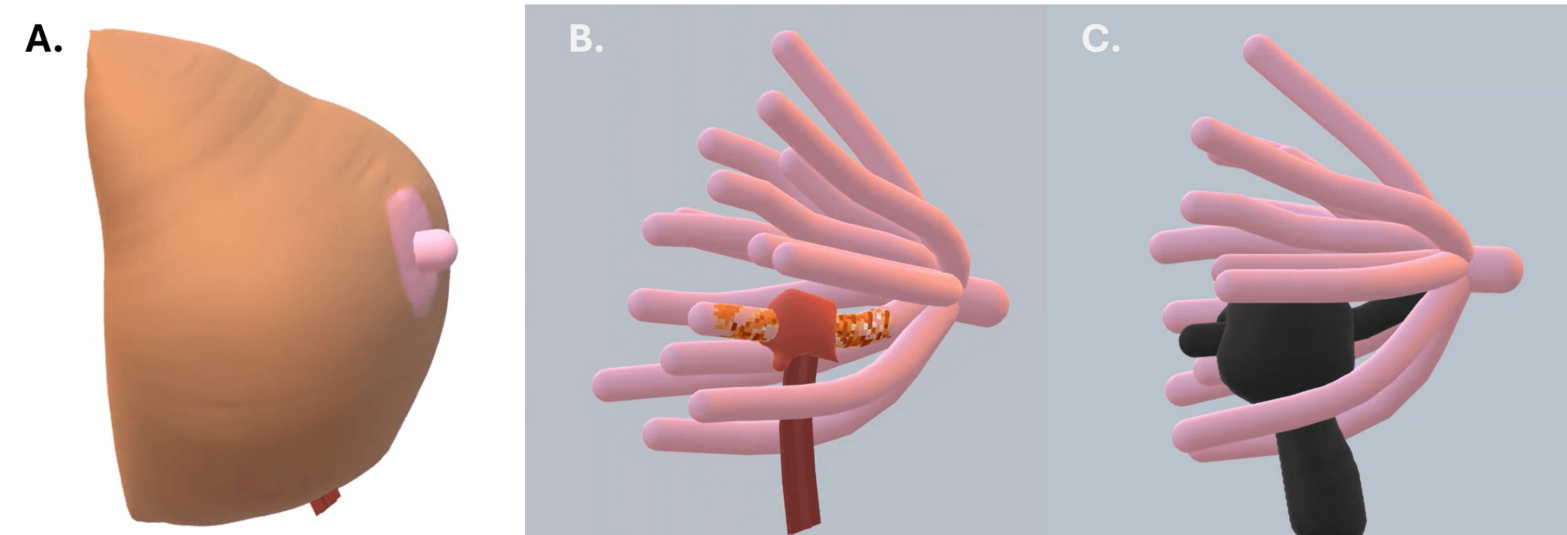
Illustration of the breast with a scar from the insertion of the percutaneous biopsy needle (A), the normal ontogenetic compartment (pink structure) and the affected by the tumor (mixed orange) and the biopsy path (red tract) (B), and surgical treatment by resection of the needle path and the ontogenetic compartment (black structure). Image credits: Eduardo de Faria Castro Fleury’

As discussed above, the extension of tumor cells along the biopsy tract leads to tumor cells in healthy adjacent ontogenetic compartments, favored by the inflammatory process resulting from the percutaneous procedure. Sometimes, tumor cells can reach the skin and subcutaneous tissue. This information should be especially relevant in the context of de-escalating surgery for breast cancer, where there could be a risk of tumor remnants after surgical treatment in the biopsy tract. The findings deserve more attention in cutaneous impairment and recurrence after surgical treatment, where breast carcinoma metastases account for 24 to 50% of cutaneous metastases (Figure 3) [11].

**Figure 4:**
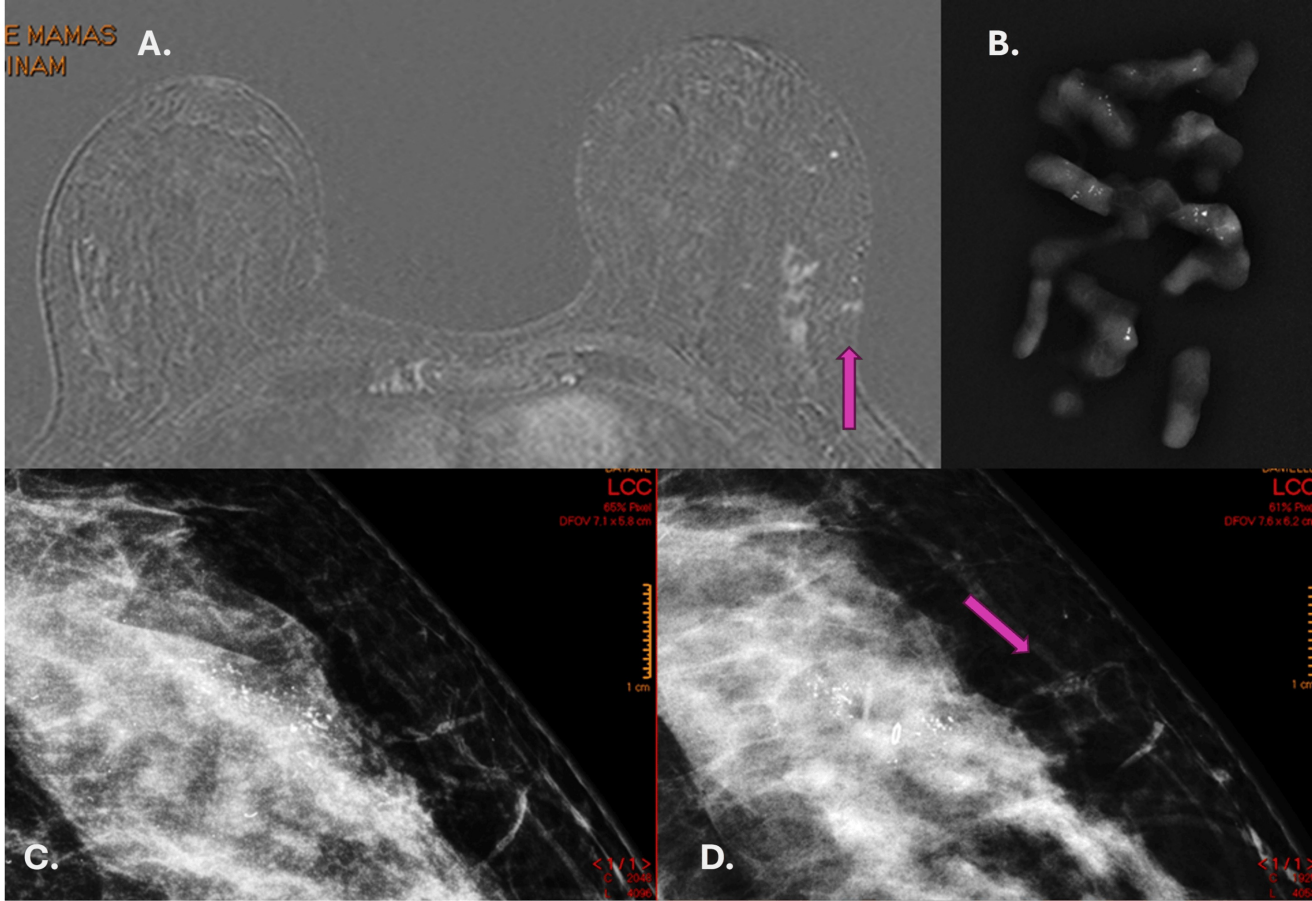
Post-contrast MRI (A), biopsy fragments (B), pre-biopsy mammogram (C), and mammogram 50 days after biopsy (D). Magnetic resonance imaging shows impairment of the biopsy path (purple arrow)(A). Vacuum-assisted biopsy specimens showing calcifications with a diagnosis of ductal carcinoma in situ (DCIS) (B). Pre-biopsy mammogram showing pleomorphic calcifications (C) and after biopsy, showing a metallic marker in the middle of the calcifications and calcifications with the same characteristics in the biopsy tract (purple arrow) (D).

As in the studies cited above, our results showed no relationship between the immunohistochemical profile and the prevalence of tumor implantation. During the study period, we observed in our clinical practice, but not included in our study protocol, one case of tumor implantation in pure ductal carcinoma in situ after performing a vacuum-assisted biopsy of suspicious calcifications (Figure 4).

**Figure 5:**
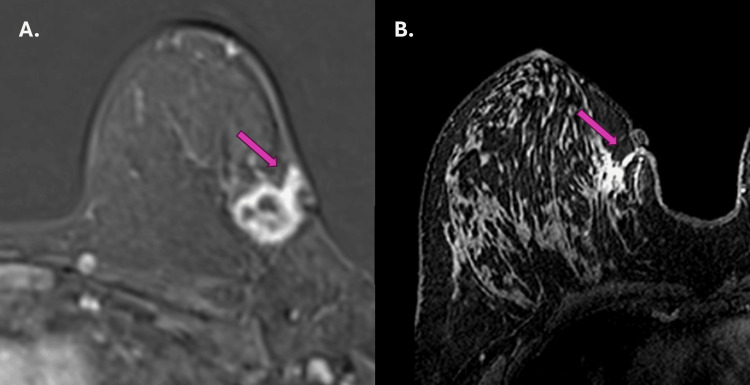
Examples of two other cases of carcinoma invasion along the biopsy path (A and B). The purple arrows (A and B) show another tumor extension through the biopsy tract in two patients from our clinical practice who were outside the study protocol.

We also show two other examples of tumor extension that we observed in our routine that were not included in our study protocol, corroborating our findings (Figure 5). Currently, we have incorporated into our practice the evaluation of the biopsy tract in patients undergoing breast carcinoma staging at magnetic resonance imaging.

According to our findings, we believe that tumor cells seeding in the biopsy tract are a consequence of the direct extension of the primary tumor into the cavity created by the percutaneous biopsy, since there is an imbalance between the defense system and the wound-healing process.

McCarty et al. argue that complicated procedures with hematoma formation can delay the resolution of the wound-healing process and favor tumor extension [9]. In this context, procedures with thinner needles, fewer fragments, and concern for adequate hemostasis with drainage of post-biopsy hematomas could minimize the chance of seeding along the biopsy tract.

Other theories justify neoplastic seeding in the setting of needle biopsy, which associates epithelial cell displacement as a precursor to seeding. Liebens et al. reported displacement of malignant cells in 22% of patients who underwent large-gauge core-needle biopsy [12]. Based on this theory, the authors propose washing the procedure needles as a preventative measure to avoid cell displacement. However, according to our findings, neoplastic seeding is due to the direct infiltration of tumor cells along the biopsy tract [13,14].

Our study has some limitations. First, it was carried out in a single institution, with only two observers. However, the study design facilitated patients' follow-up from the biopsy until the staging MRI scan. Second, due to neoadjuvant chemotherapy, the patients could only be followed up for up to 2 months, which would increase the chance of false-negative results. Other limitations were the study's retrospective nature and the small sample size. However, due to the complexity of the study design, the data allowed us to consecutively evaluate the incidence of biopsy path compromise in patients with breast cancer included in the protocol. Prospective, multicenter studies could be carried out to confirm and replicate our findings.

The presence of seeding in the biopsy tract in diagnostic biopsies in breast cancer patients is clinically relevant and can be diagnosed by MRI. It is crucial to analyze the extent of the tumor along the biopsy tract for surgical planning and safer patient management. Ignoring the spread of tumor cells through the biopsy tract in surgical planning may result in incomplete tumor resections and patients’ exposure to tumor remnants, especially to skin involvement.

## Conclusions

In conclusion, tumor seeding in the biopsy tract was found in 4.8% of patients referred for MRI scans included in our protocol. This would justify classifying these lesions in category 4 according to the BI-RADSTM lexicon. Ultrasound-guided biopsy has proven to be an efficient method for guiding the biopsy. Further investigation of this new lesion would be necessary to find the best approach for breast cancer therapeutics and management.
